# Community phylogenetic structure reveals the imprint of dispersal-related dynamics and environmental filtering by nutrient availability in freshwater diatoms

**DOI:** 10.1038/s41598-019-48125-0

**Published:** 2019-08-12

**Authors:** François Keck, Maria Kahlert

**Affiliations:** Swedish University of Agricultural Sciences, Department of Aquatic Sciences and Assessment, P. O. Box 7050, 750 07 Uppsala, Sweden

**Keywords:** Freshwater ecology, Microbial ecology, Community ecology, Limnology

## Abstract

Despite important progress, uncertainty persists regarding the ecological forces driving microbial community assembly. Here, we present the first study to use phylogenetic information to interpret the structure and diversity of diatom communities. We examined local phylogenetic divergence and beta- phylogenetic diversity in a large dataset of 595 freshwater benthic diatom communities and we investigated how this diversity is influenced by gradients in nutrients, pH, organic matter and catchment size. Overall, we found that diatom communities were phylogenetically clustered, i.e. species within communities were more closely related than expected by chance. Phylogenetic clustering was stronger in nutrient-poor environments and in sites with a small catchment area. The variation of the phylogenetic beta-diversity index was much better explained by space and environment than the variation of the taxonomic index was. Both approaches detected a significant effect of environment and space on diatom community turnover. Our results support the view that diatom communities are primarily shaped by environmental filtering, in particular by nutrient availability. Moreover, they highlight the importance of considering dispersal-related processes and the depth of phylogenetic signal in functional traits when interpreting patterns of diversity.

## Introduction

Understanding the mechanisms which determine the generation of biodiversity and the structure of biological communities is a major concern of ecology^1^. Ecologists generally agree that the structure and assembly of communities are the result of a complex combination of ecological processes that interact with each other and whose relative importance changes with spatial scale^2,3^. On the one hand, niche-related processes are deterministic and include environmental filtering and interactions among individuals (e.g. competition, predation, facilitation). On the other hand, neutral processes include stochastic events such as dispersal, speciation and extinction. Disentangling and assessing the relative importance of these processes in shaping biological communities has attracted the attention of ecologists for many years^4^. The topic continues to be heavily debated, particularly in the field of microbial ecology where the tiny size of individuals makes it difficult to track the dynamics of populations in time and space^5–8^.

In freshwater ecology, diatoms have long been used as a model taxonomic group to study microbial communities’ diversity patterns^9–11^. However, despite important progress, uncertainty persists regarding the ecological forces driving diatom community assembly. These questions have traditionally been addressed using a taxonomic perspective of diversity where taxa are regarded as independent entities. However, species are not independent entities because of their shared evolutionary history and standard statistical approaches based only on taxonomic names are limited because they cannot reflect this dependence. It is acknowledged that in order to elucidate mechanisms of ecological assembly, ecologists need to account for species differences and similarities regarding their environmental tolerances and dispersal capacities^12–14^.

Since functional diversity offers more insights into the processes at the origin of biodiversity^15,16^, a stronger emphasis has been placed on trait-based ecology in recent years^17^. However, given their large diversity and their microscopic size it is challenging to collect trait data for diatoms, and a comprehensive trait database for ecological studies is still missing^18^. To circumvent this problem, it was proposed to group diatom species into guilds or functional groups^19^. While useful to study the general structure of communities along environmental gradients^20,21^, guilds remain a very coarse classification and cannot be used to capture the multiple and subtle ecological strategy differences among species. Ecological strategies are often best represented as continuous variables^22^ and binning species into ecological guilds or functional groups can bias the analyses against detection of non-random assembly processes^14^.

Phylogenetic diversity offers a promising alternative to taxonomic and functional approaches for studying diatom ecology. Under the assumption that functional trait differences between species are correlated with the time since they diverged, phylogeny can be used as a proxy for species ecological similarity. By integrating the evolutionary history of the species, phylogenetic diversity can provide substantial insights into the ecological processes underlying community structure and composition^23,24^.

Community ecologists have been particularly interested in using methods designed to assess the phylogenetic structure of communities (i.e. divergence)^25^ to infer local ecological processes^26,27^. The observed phylogenetic divergence of a set of species within a site can be compared to the divergence in simulated null assemblages, allowing the detection of non-random patterns in community assembly. These non-random patterns of phylogenetic co-occurrence can in turn be interpreted as the result of ecological processes. If co-occurring taxa are found to be more related than in null assemblages, species are phylogenetically clustered and this is generally seen as the result of environmental filtering. Alternatively, if taxa are found to be less related than in null assemblages, species are phylogenetically over-dispersed and this is often interpreted as evidence for competitive exclusion.

In addition, it is essential to study how diversity changes across sites (beta-diversity) in order to understand the mechanisms of ecological assembly driving the structure of communities across environmental and spatial gradients^28,29^. Again, incorporating phylogenetic information into the study of compositional turnover between communities is important, as species are not independent entities^30^. Taking into account the functional variation among species through their phylogenetic relatedness is therefore a significant improvement, especially in hyperdiverse clades like diatoms where the large number of species is likely to increase the degree of functional redundancy.

While the number of phylogenetic analyses of diversity has grown exponentially in recent years, none have been conducted on diatoms. Here, we analyse the phylogenetic structure and diversity of 581 freshwater benthic diatom communities in order to investigate the ecological mechanisms that drive microbial community assembly in rivers and streams. It has long been demonstrated that niche-related processes play an important role in shaping communities of microorganisms in general^7^ and of diatoms in particular^31–33^. Our goal is to extend these results and test the hypothesis that environmental filtering is dominant but can be modulated by the environmental condition. In particular, we expect environmental filtering to be stronger in nutrient poor or acidic environments, which are known to be stressful conditions for diatoms. In order to test this hypothesis, we assess the relationship between environmental conditions and the structure of diatom communities. We also investigate the pairwise community phylogenetic structure variation along environmental and spatial gradients. Numerous recent studies of diatom beta-diversity have highlighted that communities are the result of both local factors (current ecological factors) and regional factors (origin, dispersal and extinction events). Our aim is to assess if, by including an evolutionary perspective, phylogenetic diversity can corroborate and deepen these results.

## Results

**Figure 1:**
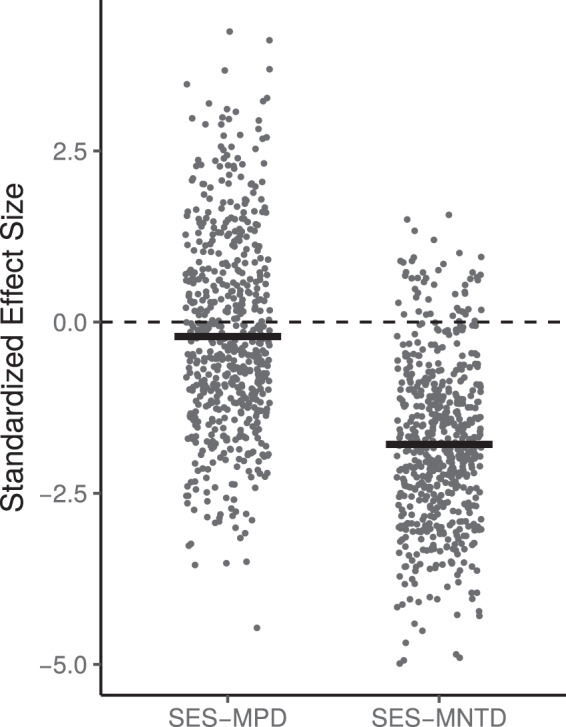
Standardized effect size of the mean pairwise distance index (SES-MPD) and the mean nearest taxon distance index (SES-MNTD) in the 595 investigated sites. The thick lines represent the means.

Across the 581 samples, the mean of the standardised mean pairwise distance index (SES-MPD) and of the standardised mean nearest taxon distance index (SES-MNTD) were both found to be significantly below zero (Fig. 1, SES-MPD mean = −0.211, t-test t = −3.533, p-value < 0.001; SES-MNTD mean = −1.786, t-test t = −36.018, p-value < 0.001), indicating a general tendency to species phylogenetic clustering within communities. Moreover, we found SES-MNTD values to be significantly lower than SES-MPD (Fig. 1, paired t-test t = −20.248, p-value < 0.001), indicating that species clustering was possibly more important near the tips of the tree.

**Figure 2:**
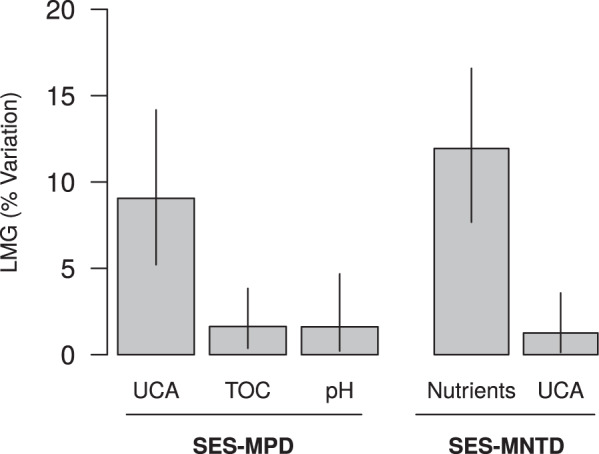
Relative importance (LMG value) of the environmental variables retained from the best fit models explaining the standardised mean pairwise distance index (SES-MPD) and the standardised mean nearest taxon distance index (SES-MNTD) variation. Vertical bars represent the 95% confidence intervals computed on the basis of 1000 bootstrap runs.

**Figure 3:**
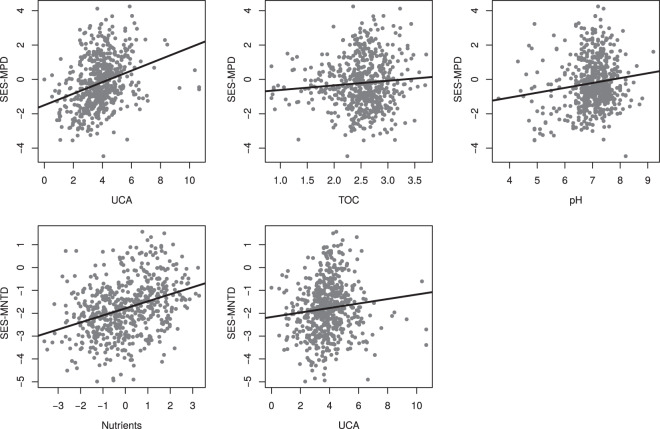
Relationship between the environmental variables retained from the best fit models and the standardised mean pairwise distance index (SES-MPD; first row) and the standardised mean nearest taxon distance index (SES-MNTD; second row). All relationships are significant (p-value < 0.05).

The best model explaining SES-MPD variation included upstream catchment area (UCA), total organic carbon (TOC) and pH (Table 1). The relative importance metric LMG identified UCA as being the most important predictor (Fig. 2). Overall, we found that SES-MPD increases with UCA, TOC and pH (Table 1, Fig. 3).

**Table 1:** Results of the best fit selected models (multiple regressions) explaining the standardised mean pairwise distance index (SES-MPD) and the standardised mean nearest taxon distance index (SES-MNTD) variation.

Model	Parameter	Estimate	Std. Error	t value	P-value
*SES-MPD*	(Intercept)	−4.4211	0.7402	−5.97	<0.001
(R^2^ = 0.12)	pH	0.2398	0.0846	2.84	0.0047
	TOC	0.501	0.1255	3.99	<0.001
	UCA	0.3314	0.0439	7.55	<0.001
*SES-MNTD*	(Intercept)	−2.1891	0.1428	−15.33	<0.001
(R^2^ = 0.13)	Nutrients	0.3105	0.0347	8.95	<0.001
	UCA	0.1046	0.0351	2.98	0.003

Models were selected by minimum AICc among all possible combinations of predictors. TOC: total organic carbon. UCA: upstream catchment area.

For SES-MNTD, the best model included UCA and nutrients (Table 1). In this case, nutrients were the most important predictor (LMG = 0.12, CI = [0.08; 0.17]). We found that both nutrients and UCA have a positive effect on SES-MNTD (Table 1, Fig. 3)

The forward selection procedure selected three environmental variables (Nutrients, pH, TOC) and 55 db-MEMs for the Jaccard index, all the environmental variables and 30 db-MEMs for the Dpw index and three environmental variables (Nutrients, pH, UCA) and 36 db-MEMs for the Dnn index. Variation partitioning (Table 2) indicated a significant effect of environment and space on turnover for all the investigated measures of beta-diversity (all p-values < 0.001). Overall, the effect of space (without environment) was found to be more important than the effect of environment (without space). The proportion of variance explained by space and environment taken together was the highest for the phylogenetic index Dnn (total R²adj = 0.42), followed by the taxonomic index Jaccard (total R²adj = 0.19). The variation of the phylogenetic index Dpw was poorly explained by the model (total R²adj = 0.04).

**Table 2:** Results of beta-diversity variation partitioning. For each index, the explained variation (R²adj) is given for the effect of environment without space (Environment|Space), the effect of space without the environment (Space|Environment), and the joint effect of environment and space (Environment ∩ Space).

Model	Fraction	df	R²adj	P-value
*Jaccard*	Environment|Space	3	0.03	<0.001
	Space|Environment	55	0.07	<0.001
	Environment ∩ Space		0.09	
	Residuals		0.81	
*Dpw*	Environment|Space	4	0.01	<0.001
	Space|Environment	30	0.02	<0.001
	Environment ∩ Space		0.01	
	Residuals		0.96	
*Dnn*	Environment|Space	3	0.10	<0.001
	Space|Environment	36	0.21	<0.001
	Environment ∩ Space		0.11	
	Residuals		0.58	

P-values of permutations tests are provided for testable fractions. Dpw: pairwise phylogenetic dissimilarity index. Dnn: pairwise nearest neighbour dissimilarity index.

## Discussion

Here we analysed a large set of diatom communities using an evolutionary perspective of diversity. At the community level we detected phylogenetic clustering which is often regarded as evidence of environmental filtering^34^. However, several factors may weaken the hypothetical links between observed patterns and on-going ecological processes^35,36^. As a consequence of this, the interpretation of non-random phylogenetic patterns is a delicate procedure which demands detailed investigation. To this end, comparing different metrics and relating their respective variation to environmental gradients can be of great help.

The SES-MPD and SES-MNTD metrics used to measure within-sites phylogenetic divergence yielded results with important differences. These differences can be explained by the fact that these two metrics are not detecting phylogenetic patterns at the same depth^37^. In theory, phylogenetic structures within communities vary according to the phylogenetic signal in functional traits and habitat association^38^, which in turn vary with the phylogenetic depth^23^. In our study, the degree of clustering was found to be stronger by SES-MNTD (Fig. 1) which, unlike SES-MPD, is a terminal metric detecting patterns near the tips of the tree. This result is consistent with our knowledge of niche evolution in diatoms, as it has been shown that the phylogenetic signal for many ecological optima is mainly located near the surface of the phylogenetic tree^39^.

Our results highlight the primary role of nutrient availability in environmental filtering of diatom communities. This was reflected by a stronger phylogenetic clustering in nutrient-poor environments (Fig. 3). Diatoms are autotrophic cells and their growth directly depends on light and nutrient supply. In low nutrient streams, adaptations providing better efficiency for nutrient uptake are strictly necessary for maintenance and ecological success. Contrary to nutrients, neither pH nor TOC were found to be important predictors of SES-MNTD, despite the fact that these two parameters are known to favour specific diatom genera (e.g. Eunotia and Frustulia for pH)^40^. However, in this dataset pH and TOC are to some extent correlated with nutrients. In particular, streams with low pH are often nutrient poor and collinearity effects could explain why pH and TOC were excluded from the SES-MNTD model.

Because ancient lineages of diatoms have diversified into sub-clades inhabiting a large variety of environments^39^, the phylogenetic signal in habitat association is assumed to be low in the deepest part of the tree. Hence, it is not surprising that we found a weak average level of structure using a basal metric like SES-MPD. However, it is worth noting that we found the strength of phylogenetic structure to be highly variable among communities (Fig. 1). This variation is mainly explained by UCA (Fig. 2, Table 1), with divergence increasing as UCA increases (Fig. 3). We suggest two possible explanations for this result. First, a small catchment upstream the sampling point is most often an indication that the stream is rather small where sampled, and is likely subject to a stronger perturbation regime which may filter morphologically adapted species. Environmental filtering would therefore be more important in the upper part of the basin, and this process could be detected with a basal metric since structural morphological features and life-forms are likely to be conserved throughout the species’ evolutionary history. Second, SES-MPD can be controlled by dispersal. As the size of the catchment area increases, the number of tributaries and the diversity of environmental conditions upstream statistically increase, thereby increasing the potential chances to recruit new lineages. Diatoms are known to disperse passively with water flows and over large distances through animals and atmospheric deposition^41^. In microbial species with high dispersal capacities like diatoms, dispersal-related dynamics can play an important role in community assembly^42^ and it has been shown that dispersal can have a strong effect on community phylogenetic structure^43^.

In general, our results do not support the idea of competitive exclusion that would select for niche differentiation. Our results can be interpreted as evidence for weak biotic interactions among coexisting species^44^. However, in the particular case of diatoms, phylogenetic distance could also be limited in explaining relative fitness differences among species, as reported in freshwater green algae^45^. It is also important to note that other factors than those we have analysed in our study, such as local water chemistry, flow disturbances and access to light are known to play an important role on benthic diatom community structure. Microbial benthic communities are organized in 3 dimensions forming complex ecosystems with vertical gradients of light and nutrients decreasing from top to bottom. In these systems (sometimes compared to microbial forests)^19,46^ erected and colony-forming species are considered to constitute the canopy while other species have developed strategies (e.g. heterotrophy, motility) to maintain themselves in the lower part of the biofilm. Similarly to terrestrial forests, the development of a river biofilm is the result of a species succession process where competition plays an important role, and in which the community composition can be largely dependent on the time since the last disturbance and the disturbance regime. However, high-resolution data on disturbances and light penetration through biofilms are rarely available in large scale diatom assessments. Hence, the study of phylogenetic community structure dynamics during diatom successional changes would require further *in situ* colonisation controlled experiments.

The beta-diversity analyses revealed a significant effect of environment and space on diatom community turnover, regardless of the metric used (Table 2). This result was expected, as many recent studies have highlighted the joint effect of niche related processes and dispersal limitation in controlling diatom community taxonomic structure^33,42,47,48^. Nonetheless, it is important to note that the variation in Dnn was much better explained by space and environment than the taxonomic beta-diversity metric (Jaccard). This result can be attributed to the fact that phylogenetic diversity (here Dnn) can achieve a better ecological realism than taxonomic beta-diversity, precisely because it incorporates information on species similarities. In this case, a high functional redundancy is likely to generate a high turnover between closely related diatom species which is best modelled by a phylogenetic metric of diversity.

Similarly to local phylogenetic divergence, phylogenetic beta-diversity is highly dependent on the strength and location of phylogenetic signal in habitat association. Thus, Dpw which is a basal metric^37,49^, was very poorly explained by the environmental matrix as a consequence of the low phylogenetic signal in diatom ecological niche in the deeper part of the tree. In addition, the amount of variance in Dpw explained by spatial predictors was found to be very low, indicating a weak phylogeographic signal. Again, the strong dispersal capacities of microscopic organisms can explain this observation. At the investigated scale, the dispersal rate of diatoms is high enough to obscure any geographical signature of historical colonization or local diversification.

Our study offers new insight into the community assembly of freshwater diatoms and confirms the ability of phylogenetic approaches to shed new light on long-standing questions in microbial ecology. Previous works have approached ecological questions in benthic diatoms by using taxonomic ranks as a surrogate for phylogenetic information^44,50^. However, molecular phylogenies constitute a significant improvement over taxonomy because identically ranked taxa are often not comparable in terms of age and diversification rate^51^. The phylogenetic tree used in this study reflects more closely the evolutionary history of diatoms than a taxonomy-based approach would do, and provides a good estimate of the relative age of the genera. Still, a large number of species have been inserted on the basis of taxonomy and the nested phylogenetic relationships below the genus level remain poorly resolved. It should also be noted that in this study, the phylogenetic information was collected separately from the biological data. The diatom community compositions were obtained through classical morphology-based microscopy, but the strength of these data is that they were produced by a handful of trained diatomists and they were all intercalibrated and harmonized. However, our approach of mapping taxa identified through microscopy onto a molecular phylogeny can be limited by discrepancies between morpho-taxonomy and genetic diversity. We can therefore expect that future studies will take advantage of advances in diatom sequencing and phylogenetic reconstruction to study environmental filtering and competitive exclusion at the finest resolution possible. Further investigations will also be necessary to combine phylogeny with trait data (upon availability) in order to provide an integrated perspective of the mechanisms driving diatom ecology and community assembly. It will be particularly interesting to contrast which traits explain deep and recent phylogenetic structures in diatom communities. Finally, the question of spatial scale, which is also important in understanding biological diversity across time and space, should be investigated in combination with phylogeny in future studies.

## Materials and Methods

### Data

A total of 581 diatom samples were collected across lotic systems in Sweden (Supplementary Fig. S1) between 1998 and 2010 (between August and October) and prepared following standard protocols^52,53^. For each sample, at least 400 valves were counted under microscope, and identified to the lowest taxonomic level possible using the literature in the Swedish standard^54^. Identification was performed by only a few Swedish analysts, all harmonized to conventions adopted by the Nordic-Baltic Network for Benthic Algae in Freshwater^55^, meaning that the variation in diatom species lists was not larger between than within analysts^56^.

We considered four environmental variables, based on their availability and recognized importance for diatom communities: pH, total organic carbon (TOC), nutrients (composite variable including nitrogen and phosphorus) and upstream catchment area (UCA). For each environmental variable we computed the mean value of the records available for the 12 months period preceding the biological sampling. Both TOC and UCA were log-transformed prior to analyses. Nitrogen and phosphorus concentration were found to be highly correlated which can be problematic for inference analyses. Therefore, they were pooled into one single variable reflecting nutrient availability. This variable was calculated as the site projection on the first component of a principal component analysis (PCA) including log-transformed total nitrogen and log-transformed total phosphorus concentrations.

### Phylogeny

We used the time-calibrated phylogeny of diatoms published by Nakov *et al*.^57^. This tree of 1151 taxa is the most inclusive diatom species phylogeny to date. It was reconstructed by maximum likelihood using 11 genes (18 S rRNA, 28 S rRNA, 16 S rRNA, *atp*B, *psa*A, *psa*B, *psb*A, *psb*C, *rbc*L, *cob* and *cox*I) and 38 calibration points (see^57^ for details). The phylogeny included 193 taxa of the community dataset. We grafted missing taxa to the most recent common ancestor of all members sharing the lowest taxonomic level available in the tree. Missing varieties (67) were inserted at species-level and missing species (515) were inserted at genus-level. This way, each inserted taxon is separated from the other on the basis of its taxonomic name and is replaced on an independent branch in the tree. Missing taxa belonging to genera that were not available in the tree were not used in the analyses (98). Therefore, the analyses were conducted on 775 taxa. The complete list of species included in the analyses is available as Supplementary Methods S1.

### Phylogenetic community structure

To measure the phylogenetic structure of the communities we calculated two statistics: the mean pairwise distance index (MPD) and the mean nearest taxon distance index (MNTD). Both MPD and MNTD are classical measures of phylogenetic divergence and can be used to assess whether local communities are non-randomly structured^25,26^. However, they differ in their sensitivity to phylogenetic depth^37,58^. The MPD index is a basal metric which is sensitive to deep phylogenetic structures, whereas the MNTD index is a terminal metric which is sensitive to structures near the tips of the tree. Because these measures of phylogenetic diversity are not statistically independent from the species richness, we calculated a standardized effect size of each metric (SES-MPD and SES-MNTD) against a distribution of 1000 null values computed by shuffling the tip labels in the tree.

To examine changes in community structure across the environmental gradients, we used linear models to regress SES-MPD and SES-MNTD against environmental variables. In each case, the best fit model was selected based on the minimum AICc score. The relative importance of the variables in the best models was assessed using the average of sequential explained variances over all possible orderings of regressors, noted LMG^59^.

### Phylogenetic beta-diversity

For each pair of sites, we calculated a taxonomic dissimilarity index and two phylogenetic dissimilarity indices. We used the Jaccard index to measure the taxonomic turnover in diatom composition among sites and the intercommunity MPD (Dpw) and MNTD (Dnn) indices to measure the phylogenetic beta-diversity.

We used distance based redundancy analysis (db-RDA) to relate the variation in the different diversity indices to the environmental gradients. Finally, variation partitioning was used to examine the relative importance of space and environment in explaining taxonomic and phylogenetic turnover among sites. In variation partitioning, space was modelled using distance-based Moran eigenvector maps (db-MEM)^60,61^. We extracted db-MEM exhibiting a positive correlation and performed a forward selection procedure with double stopping criterion^62^ to limit the number of variables included in each model.

### Software

Analyses were performed in R v3.3.1. Taxonomic diversity analyses were conducted with vegan^63^ and phylogenetic diversity analyses with picante^64^. Relative importance metrics (LMG) for linear regression were computed with relaimpo^65^. We computed db-MEM with adespatial^66^. Multivariate analyses were conducted with vegan.

## Supplementary information

List of species included in the study

Maps of sampling sites

## Data Availability

Data were extracted from the open access Swedish national database (http://miljodata.slu.se/).

## References

[1] MacArthur, R. H. *Geographical ecology: patterns in the distribution of species*. (Princeton University Press, 1972).

[2] Levin SA (1992). The Problem of Pattern and Scale in Ecology: The Robert H. MacArthur Award Lecture. Ecology.

[3] Ricklefs RE (2004). A comprehensive framework for global patterns in biodiversity. Ecol. Lett..

[4] Chase JM, Myers JA (2011). Disentangling the importance of ecological niches from stochastic processes across scales. Philos. Trans. R. Soc. Lond. B Biol. Sci..

[5] Fenchel T, Finlay BJ (2004). The Ubiquity of Small Species: Patterns of Local and Global Diversity. BioScience.

[6] Martiny JBH (2006). Microbial biogeography: putting microorganisms on the map. Nat. Rev. Microbiol..

[7] Van der Gucht K (2007). The power of species sorting: Local factors drive bacterial community composition over a wide range of spatial scales. Proc. Natl. Acad. Sci..

[8] Lindström ES, Langenheder S (2012). Local and regional factors influencing bacterial community assembly. Environ. Microbiol. Rep..

[9] Potapova MG, Charles DF (2002). Benthic diatoms in USA rivers: distributions along spatial and environmental gradients. J. Biogeogr..

[10] Soininen J, Paavola R, Muotka T (2004). Benthic diatom communities in boreal streams: community structure in relation to environmental and spatial gradients. Ecography.

[11] Soininen J, Jamoneau A, Rosebery J, Passy SI (2016). Global patterns and drivers of species and trait composition in diatoms. Glob. Ecol. Biogeogr..

[12] MacArthur R, Levins R (1967). The Limiting Similarity, Convergence, and Divergence of Coexisting Species. Am. Nat..

[13] Westoby M, Wright IJ (2006). Land-plant ecology on the basis of functional traits. Trends Ecol. Evol..

[14] Kraft NJB, Ackerly DD (2010). Functional trait and phylogenetic tests of community assembly across spatial scales in an Amazonian forest. Ecol. Monogr..

[15] Shipley B, Vile D, Garnier É (2006). From Plant Traits to Plant Communities: A Statistical Mechanistic Approach to Biodiversity. Science.

[16] Kraft NJB, Valencia R, Ackerly DD (2008). Functional Traits and Niche-Based Tree Community Assembly in an Amazonian. Forest. Science.

[17] McGill BJ, Enquist BJ, Weiher E, Westoby M (2006). Rebuilding community ecology from functional traits. Trends Ecol. Evol..

[18] Tapolczai K, Bouchez A, Stenger-Kovács C, Padisák J, Rimet F (2016). Trait-based ecological classifications for benthic algae: review and perspectives. Hydrobiologia.

[19] Passy SI (2007). Diatom ecological guilds display distinct and predictable behavior along nutrient and disturbance gradients in running waters. Aquat. Bot..

[20] Berthon V, Bouchez A, Rimet F (2011). Using diatom life-forms and ecological guilds to assess organic pollution and trophic level in rivers: a case study of rivers in south-eastern France. Hydrobiologia.

[21] Stenger-Kovács C, Lengyel E, Crossetti LO, Üveges V, Padisák J (2013). Diatom ecological guilds as indicators of temporally changing stressors and disturbances in the small Torna-stream, Hungary. Ecol. Indic..

[22] Westoby M, Falster DS, Moles AT, Vesk PA, Wright IJ (2002). Plant Ecological Strategies: Some Leading Dimensions of Variation between Species. Annu. Rev. Ecol. Syst..

[23] Cavender-Bares J, Kozak KH, Fine PVA, Kembel SW (2009). The merging of community ecology and phylogenetic biology. Ecol. Lett..

[24] Mouquet N (2012). Ecophylogenetics: advances and perspectives. Biol. Rev..

[25] Tucker CM (2017). A guide to phylogenetic metrics for conservation, community ecology and macroecology. Biol. Rev..

[26] Webb CO, Ackerly DD, McPeek MA, Donoghue MJ (2002). Phylogenies and community ecology. Annu. Rev. Ecol. Syst..

[27] Cavender-Bares J, Ackerly DD, Baum DA, Bazzaz FA (2004). Phylogenetic overdispersion in Floridian oak communities. Am. Nat..

[28] Whittaker RH (1972). Evolution and Measurement of Species Diversity. Taxon.

[29] Anderson MJ (2011). Navigating the multiple meanings of β diversity: a roadmap for the practicing ecologist. Ecol. Lett..

[30] Graham CH, Fine PVA (2008). Phylogenetic beta diversity: linking ecological and evolutionary processes across space in time. Ecol. Lett..

[31] Patrick, R. Ecology of Freshwater Diatoms - Diatom Communities. In *The Biology of Diatoms* (ed. Werner, D.) 284–332 (University of California Press, 1977).

[32] Pan Y, Stevenson RJ, Hill BH, Kaufmann PR, Herlihy AT (1999). Spatial Patterns and Ecological Determinants of Benthic Algal Assemblages in Mid-Atlantic Streams, Usa. J. Phycol..

[33] Soininen J (2007). Environmental and Spatial Control of Freshwater Diatoms—a Review. Diatom Res..

[34] Webb CO (2000). Exploring the Phylogenetic Structure of Ecological Communities: An Example for Rain Forest Trees. Am. Nat..

[35] Mayfield MM, Levine JM (2010). Opposing effects of competitive exclusion on the phylogenetic structure of communities. Ecol. Lett..

[36] Gerhold P, Cahill JF, Winter M, Bartish IV, Prinzing A (2015). Phylogenetic patterns are not proxies of community assembly mechanisms (they are far better). Funct. Ecol..

[37] Swenson NG (2011). Phylogenetic Beta Diversity Metrics, Trait Evolution and Inferring the Functional Beta Diversity of Communities. PLoS ONE.

[38] Losos JB (2008). Phylogenetic niche conservatism, phylogenetic signal and the relationship between phylogenetic relatedness and ecological similarity among species. Ecol. Lett..

[39] Keck F, Rimet F, Franc A, Bouchez A (2016). Phylogenetic signal in diatom ecology: Perspectives for aquatic ecosystems biomonitoring. Ecol. Appl..

[40] Kahlert M, Gottschalk S (2014). Differences in benthic diatom assemblages between streams and lakes in Sweden and implications for ecological assessment. Freshw. Sci..

[41] Kristiansen J (1996). Dispersal of freshwater algae — a review. Hydrobiologia.

[42] Heino J (2010). Geographical patterns of micro-organismal community structure: are diatoms ubiquitously distributed across boreal streams?. Oikos.

[43] Kembel SW (2009). Disentangling niche and neutral influences on community assembly: assessing the performance of community phylogenetic structure tests. Ecol. Lett..

[44] Bottin M, Soininen J, Alard D, Rosebery J (2016). Diatom Cooccurrence Shows Less Segregation than Predicted from Niche Modeling. PLoS ONE.

[45] Narwani A, Alexandrou MA, Oakley TH, Carroll IT, Cardinale BJ (2013). Experimental evidence that evolutionary relatedness does not affect the ecological mechanisms of coexistence in freshwater green algae. Ecol. Lett..

[46] Round, F. E., Crawford, R. M. & Mann, D. G. *The diatoms: biology and morphology of the genera*. (Cambridge University Press, 1990).

[47] Vyverman W (2007). Historical Processes Constrain Patterns in Global Diatom Diversity. Ecology.

[48] Verleyen E (2009). The importance of dispersal related and local factors in shaping the taxonomic structure of diatom metacommunities. Oikos.

[49] Jin LS, Cadotte MW, Fortin M-J (2015). Phylogenetic turnover patterns consistent with niche conservatism in montane plant species. J. Ecol..

[50] Passy SI, Bottin M, Soininen J, Hillebrand H (2016). Environmental filtering and taxonomic relatedness underlie the species richness–evenness relationship. Hydrobiologia.

[51] Nakov T, Beaulieu JM, Alverson AJ (2018). Insights into global planktonic diatom diversity: The importance of comparisons between phylogenetically equivalent units that account for time. ISME J..

[52] European Committee for Standardization. *Water quality - Guidance standard for the routine sampling and pretreatment of benthic diatoms from rivers*. (2003).

[53] European Committee for Standardization. *Water quality - Guidance standard for the identification, enumeration and interpretation of benthic diatom samples from running waters*. (2004).

[54] Havs och Vattenmyndigheten. *Programområde: Sötvatten. Undersökningstyp: Påväxt i sjöar och vattendrag – kiselalgsanalys* (2016).

[55] Kahlert, M. & Albert, R.-L. NorBAF – The Nordic-Baltic Network for Benthic Algae in Freshwater. Available at, http://www.norbaf.net/ (2005).

[56] Kahlert M (2009). Harmonization is more important than experience—results of the first Nordic–Baltic diatom intercalibration exercise 2007 (stream monitoring). J. Appl. Phycol..

[57] Nakov T, Beaulieu JM, Alverson AJ (2018). Accelerated diversification is related to life history and locomotion in a hyperdiverse lineage of microbial eukaryotes (Diatoms, Bacillariophyta). New Phytol..

[58] Swenson, N. G. *Functional and Phylogenetic Ecology in R*. (Springer, 2014).

[59] Lindeman, R. H., Merenda, P. F. & Gold, R. Z. *Introduction to bivariate and multivariate analysis*. (Scott, Foresman, 1980).

[60] Borcard D, Legendre P (2002). All-scale spatial analysis of ecological data by means of principal coordinates of neighbour matrices. Ecol. Model..

[61] Dray S, Legendre P, Peres-Neto PR (2006). Spatial modelling: a comprehensive framework for principal coordinate analysis of neighbour matrices (PCNM). Ecol. Model..

[62] Blanchet FG, Legendre P, Borcard D (2008). Forward Selection of Explanatory Variables. Ecology.

[63] Oksanen, J. *et al*. *vegan: Community Ecology Package*. (2016).

[64] Kembel SW (2010). Picante: R tools for integrating phylogenies and ecology. Bioinformatics.

[65] Groemping, U. Relative Importance for Linear Regression in R: The Package relaimpo. *J. Stat. Softw*. **17** (2007).

[66] Dray, S. *et al*. *adespatial: Multivariate Multiscale Spatial Analysis* (2016).

