# Supplementary material for: Community phylogenetic structure reveals the imprint of dispersal-related dynamics and environmental filtering by nutrient availability in freshwater diatoms
Source: Sci Rep. 2019 Aug 12;9:11590. doi: 10.1038/s41598-019-48125-0 (PMC6691006; doi:10.1038/s41598-019-48125-0)

**Community phylogenetic structure reveals the imprint of dispersal-related dynamics and environmental filtering by nutrient availability in freshwater diatoms**

François Keck (francois.keck@gmail.com) and Maria Kahlert (maria.kahlert@slu.se)

Swedish University of Agricultural Sciences, Department of Aquatic Sciences and

Assessment, P. O. Box 7050, 750 07 Uppsala, Sweden

**Supplementary Figure S1.** Map of Sweden showing the locations of the 581 diatom sampling sites.


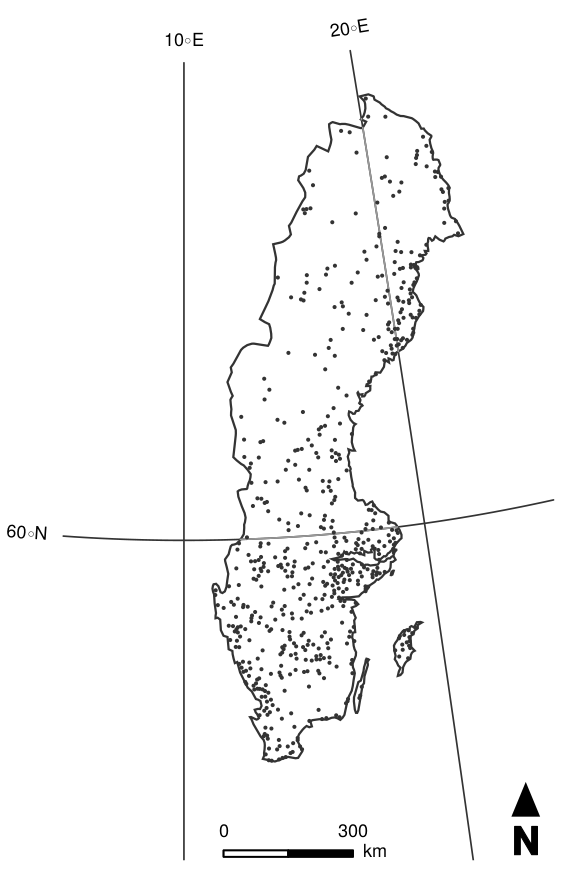

Supplement: Supplementary file 1 — List of species included in the study [file 41598_2019_48125_MOESM1_ESM.docx]
